# Intra-Laboratory Evaluation of Luminescence Based High-Throughput Serum Bactericidal Assay (L-SBA) to Determine Bactericidal Activity of Human Sera against *Shigella*

**DOI:** 10.3390/ht9020014

**Published:** 2020-06-08

**Authors:** Omar Rossi, Eleonora Molesti, Allan Saul, Carlo Giannelli, Francesca Micoli, Francesca Necchi

**Affiliations:** GSK Vaccines Institute for Global Health s.r.l (GVGH), Siena 53100, Italy; omar.x.rossi@gsk.com (O.R.); eleonora.molesti@gmail.com (E.M.); allan.saul@honorary.burnet.edu.au (A.S.); carlo.x.giannelli@gsk.com (C.G.); francesca.x.micoli@gsk.com (F.M.)

**Keywords:** *Shigella*, Generalized Modules for Membrane Antigens (GMMA), vaccine, Serum Bactericidal Assay (SBA), human, luminescence, functional assay

## Abstract

Despite the huge decrease in deaths caused by *Shigella* worldwide in recent decades, shigellosis still causes over 200,000 deaths every year. No vaccine is currently available, and the morbidity of the disease coupled with the rise of antimicrobial resistance renders the introduction of an effective vaccine extremely urgent. Although a clear immune correlate of protection against shigellosis has not yet been established, the demonstration of the bactericidal activity of antibodies induced upon vaccination may provide one means of the functionality of antibodies induced in protecting against *Shigella*. The method of choice to evaluate the complement-mediated functional activity of vaccine-induced antibodies is the Serum Bactericidal Assay (SBA). Here we present the development and intra-laboratory characterization of a high-throughput luminescence-based SBA (L-SBA) method, based on the detection of ATP as a proxy of surviving bacteria, to evaluate the complement-mediated killing of human sera. We demonstrated the high specificity of the assay against a homologous strain without any heterologous aspecificity detected against species-related and non-species-related strains. We assessed the linearity, repeatability and reproducibility of L-SBA on human sera. This work will guide the bactericidal activity assessment of clinical sera raised against *S. sonnei*. The method has the potential of being applicable with similar performances to determine the bactericidal activity of any non-clinical and clinical sera that rely on complement-mediated killing.

## 1. Introduction

Diarrheal diseases, such as shigelloses and salmonellosis, are the second leading cause of death worldwide, resulting in millions of deaths per year, mostly in developing countries [1]. *Shigella* is major cause of sustained endemic bacterial diarrhea, especially in low- and middle-income countries where accessibility to clean water is restricted. Although the improvement of hygienic conditions in the last decade has dramatically reduced the burden of the disease, *Shigella* is still responsible for more than 200,000 deaths, with a third of them being young children [1]. On top of the deaths in endemic countries, enteric diseases are causing diarrhea among travelers and military personnel in developed countries, further increasing the burden and the economic and social impact. Therefore, the huge morbidity and mortality of the disease coupled with the rise of antimicrobial resistance [2] render the introduction of a vaccine a priority for public health. Although several approaches have been tried during the years by several groups worldwide, no vaccines are yet licensed. Among the different approaches used to produce *Shigella* vaccines, many of the candidate vaccines target the serotype-specific O-Antigen (OAg) part of the lipopolysaccharide (LPS), as OAg has been identified as a key antigen recognized by the immune system after natural infection [3]. In fact, although multiple immune mechanisms may provide protection against *Shigella* and are not yet fully elucidated, it is well established that antibodies directed to OAg can fix complement and kill target bacteria in a serotype-specific manner [3,4]. Genus *Shigella* is composed by four subgroups (*S. flexneri*, *S. sonnei*, *S. dysenteriae*, and *S. boydii*) and each of them, with the exception of *S. sonnei*, is composed by different serotypes, for a total of over 50 different serotypes based on the structure of the OAg, with relative prevalence of serotypes changing geographically and over time [5]. As LPS antibody production can confer protection from homologous serotypes, a multivalent *Shigella* vaccine is necessary to induce antibodies to LPS OAg from multiple serotypes in order to confer broad protection.

Several approaches are currently in development to deliver the O-antigen to the immune system, including whole-cell attenuated bacteria [6], vaccines in which the O-antigens are chemically- [7] or bio-conjugated to carrier proteins [8], synthetic vaccine conjugates [9], and Generalized Modules for Mebrane Antigens (GMMA)-based vaccines [10]. GMMA are outer membrane exosomes released from Gram-negative bacteria, genetically modified to induce hyperblebbing and to reduce the reactogenic potential of lipid A [11,12]. GMMA are easy and inexpensive to produce and highly immunogenic [10,13,14,15,16]. The most advanced GMMA-based vaccine, 1790GAHB [10], has been tested in phase I and IIa clinical trials, conducted in European [17] and endemic sites [18], and has been demonstrated to be well-tolerated, immunogenic, and able to induce a strong anamnestic response after boosting [19]. 

On top of vaccine immunogenicity, traditionally assessed through the measurement of serum antibodies via antigen specific ELISA, the functionality of antibodies raised also needs to be documented. Although no correlate of protection has been yet established for *Shigella*, different approaches to assess the functionality of antibodies as an immunological endpoint against *Shigella* have been evaluated and have been recently reviewed [20]. Among them, the serum bactericidal assay (SBA) constitutes the method of choice to measure complement-mediated bacterial killing. SBA has been accepted as an in vitro correlate of protection for the evaluation of the immunogenicity of other bacterial vaccines, such as cholera [21] and meningococcal disease [22]. 

The working principle of SBA relies on reconstituting the in vitro conditions in which antibodies recognize antigen on the surface of target bacterium and bind to the exogenous complement, activating the classical pathway, thus resulting in bacteriolysis and the death of the target organism. The major problem with traditional SBA is that it relies on plating and counting the target bacteria. Therefore, conventional SBA has been often considered time-consuming and labor-intensive for screening large datasets and clinical samples [22]. However, efforts have been made in order to increase the analytical throughput of the assay, resulting in the development of both conventional [23] and non-conventional [24,25] high-throughput SBA. We have previously demonstrated the usefulness of a high-throughput SBA method based on luminescence (L-SBA) as a survival readout for several pathogens (including *S. flexneri* and *S. sonnei*, *Salmonella* Typhimurium, Enteritidis and Paratyphi A) using both animal [24] and human sera [26]. The number of viable bacterial cells surviving the complement-mediated antibody dependent killing is quantified by measuring their metabolic ATP. After bacteria lysis, ATP becomes available to trigger a luciferase-mediated reaction, resulting in a measurable luminescence signal. In L-SBA the level of luminescence detected is proportional to the number of living bacteria present in the assay wells, which is inversely proportional to the level of functional antibodies present in the serum [24]. The result of the assay is the IC50, the dilution of sera able to kill half of the bacteria present in the assay, thus representing the SBA titer of the sera. We have already demonstrated the possibility of using the L-SBA to determine the bactericidal activity of sera raised against *S. sonnei* GMMA in pre-clinical models [14]. Here we present the further development of this method, showing its full characterization using human sera, and in particular using sera raised against a *S. sonnei* GMMA-based vaccine (1790GAHB) as model. We have characterized the method intra-laboratory by assessing its specificity, linearity, and precision. 

The L-SBA assay described here is a useful tool for measuring functional antibodies elicited not only by GMMA-based vaccines, but in general to assess the *Shigella*-specific functional antibodies in vitro, and potentially of all vaccines that induce antibodies capable of complement-mediated killing, either from preclinical and clinical sera. 

## 2. Materials and Methods

### 2.1. Bacterial Strains and Reagents

Working aliquots of *S. sonnei virG*::*cat* [27], a strain with stabilized major virulence plasmid (pSS), thus resulting in a stabilized OAg expression in vitro when grown in the presence of antibiotic, stored frozen at −80 °C in 20% glycerol stocks were grown overnight (16–18 h) at 37 °C in a Luria Bertani (LB) medium supplemented with 20 µg/mL chloramphenicol, stirring at 180 rpm. The overnight bacterial suspension was then diluted in fresh LB medium supplemented with 20 µg/mL of chloramphenicol to an optical density at 600 nm (OD_600_) of 0.05 and incubated at 37 °C with 180 rpm agitation in an orbital shaker, until reaching OD_600_ of 0.22 +/− 0.02. Baby (3- to 4-week-old) rabbit complement (BRC) was purchased from Cederlane, stored in 10 mL frozen aliquots, and thawed immediately prior use. Phosphate Buffer Saline at pH 7 (PBS) was used for serum and bacteria dilutions. LPS was extracted from *S. sonnei* by hot phenol extraction as previously reported [10], whereas OAg was extracted from *S. flexneri* 1b, 2a 3a and from *Salmonella* Typhimurium by direct acid hydrolysis, as previously reported [14]. All extracted polysaccharides were fully characterized in terms of sugar content, protein and nucleic acid impurities by a combination of analytical techniques, including High-Performance Liquid Size Exclusion Chromatography [28], micro-BCA and absorption at 260 nm as previously reported [14].

### 2.2. Serum Samples

The human serum tested was an anti-human *S. sonnei* IgG standard serum (NVGH2863) that was created by pooling sera from adult subjects immunized with 1790 GAHB in non-endemic European populations [17]. NVGH2863 has been already used as standard serum for *S. sonnei* LPS IgG assessment by ELISA [17,18,19]. Frozen 50 µL working aliquots of the serum were stored at −80 °C until use. In the setup of the experiments, a standard serum obtained from mice immunized with 1790GAHB (NVGH1894) [24] was also included. All samples tested in SBA were previously Heat Inactivated (HI) at 56 °C for 30 min to remove endogenous complement activity. Various aliquots of HI NVGH2863 serum have been used and treated as described below to determine the different assay parameters. 

Samples used to assess repeatability and intermediate precision: each sample consists on the same HI NVGH2863 serum; 12 identical samples were assayed each day by two operators and the assay was repeated on three different days by each of the two operators independently (72 samples in total, 36 per operator, 12 on each day).

Samples used to assess limit of detection and limit of quantitation: HI NVGH2863 was diluted 10 times *v*:*v* in PBS to generate a sample with low but detectable SBA titer (expected IC50 to be around 100). Twelve identical NVGH2863 prediluted serum samples were assayed on the same day by the same operator. HI NVGH2863 was also diluted 100 times *v*:*v* in PBS to generate a sample with a low but detectable SBA titer (expected IC50 to be around 10) performing the L-SBA testing the sera starting from 1:4 dilution. For this latter test, the L-SBA was performed under the same conditions described below for the sera tested 1:30, but adapting the volume of reagents to maintain the same % of BRC, concentration of bacteria, and the total reaction volume in the assay well. Twelve identical NVGH2863 prediluted serum samples were assayed on the same day by the same operator.

Samples used to assess linearity: HI NVGH2863 serum was assayed neat or diluted 2, 4, 8, 16, 32-fold (*v*:*v*) with PBS prior performing the assay; samples were prepared independently by two operators on the same day, with each sample assayed twice by the same operator on the same day (4 IC50 obtained for each dilution, 2 IC50 by each operator).

Samples used to assess specificity: two sets of samples were prepared to assess the homologous and heterologous specificity of the assay using HI NVGH2863 serum diluted 1:1 (*v*:*v*) in PBS alone or PBS supplemented with different quantity of homologous or heterologous purified polysaccharides. In the first experiment HI NVGH2863 serum was spiked with homologous (*S. sonnei*) purified LPS at different final concentrations (50, 20, 5, 1, 0.1 µg/mL respectively) and compared with a sample spiked 1:1 with PBS alone, incubated overnight (16–18 h) at 4 °C shaking at 200 rpm in an orbital shaker, prior to being tested. Each spiked sample was assayed in duplicate by the same operator on the same day. The lowest concentration of LPS between the ones tested able to inhibit >70% the IC50 was then used in a second experiment to determine the heterologous specificity. In the second experiment, HI NVGH2863 serum diluted 1:1 (*v*:*v*) in PBS supplemented with *S. flexneri* 1b, *S. flexneri* 2a, *S. flexneri* 3a OAg (heterologous but from the same species) or *Salmonella* Typhimurium OAg (heterologous from a different species) was prepared and assayed in comparison to sample preincubated overnight with an equal volume of PBS alone (undepleted) and a sample preincubated with *S. sonnei* LPS (to confirm homologous specificity). All samples were incubated overnight (16–18 h) at 4 °C shaking at 200 rpm in an orbital shaker prior being tested. Each spiked sample was assayed in duplicate by the same operator on the same day. 

### 2.3. Luminescent-SBA (L-SBA)

A serum bactericidal assay based on luminescent readout (L-SBA) was performed in 96-well round bottom sterile plates (Corning)―the SBA plate―by incubating different dilutions of HI test sera in PBS in the presence of exogenous complement (BRC) and bacteria. The HI sera were serially diluted in PBS in the SBA plate (10 µL/well). The starting dilution of each serum in the assay was 1:30 (final dilution), followed by 3-fold dilution steps up to 7 dilution points, plus 1 control well with no sera added that represented control for non-specific complement killing as well as a sample diluted infinite-fold. Up to 12 different sera can be assayed within each SBA plate. Log-phase cultures (OD_600_ = 0.22 ± 0.02) were prepared as described above and diluted to approximately 1 × 10^6^ Colony Forming Unit (CFU)/mL in PBS. An adequate volume of reaction mixture containing the target bacterial cells (10 µL/well) and BRC (20 µL/well) as an external source of complement in PBS medium (60 µL/well) was prepared; 90 µL/well of reaction mixture were added to each well of the SBA plate containing HI serum dilutions (final reaction volume 100 µL), mixed and incubated for 3 h at 37 °C. At the end of the incubation, the SBA plate was centrifuged at room temperature for 10 min at 4000× *g*. The supernatant was discarded to remove ATP derived from dead bacteria and SBA reagents. The remaining live bacterial pellets were resuspended in PBS, transferred in a white round-bottom 96-well plate (Greiner) and mixed 1:1 *v*:*v* with BacTiter-Glo Reagent (Promega, Southampton, United Kingdom). The reaction was incubated for 5 min at room temperature on an orbital shaker, and the luminescence signal measured by a luminometer (Synergy HT, Biotek, Swindon, UK).

### 2.4. Calculations

The level of luminescence detected is directly proportional to the number of living bacteria present in the wells, which is inversely proportional to the level of functional antibodies present in the serum [24]. A 4-parameter non-linear regression was applied to the raw luminescence (no normalization of data was applied) obtained for all the sera dilutions tested for each serum; an arbitrary serum dilution of 10^15^ was assigned to the well containing no sera. Fitting was performed by weighting the data for the inverse of luminescence^2 and constraining the curves to have a bottom between 0 and 400 CPS. 400 CPS is the approximate value corresponding to the lowest luminescence detected at T180 for sera in all the wells in which bacteria are killed (300 CPS) plus the SD of luminescence detected on those wells (100). To validate the assay plate, the average luminescence at T180 detected in wells with no sera had to be at least 5-fold higher with respect to luminescence detected at T0. To validate the dilution series, the highest luminescence detected in the dilution series at T180 had to be at least 0.7-fold the luminescence detected in the control well with no sera added. The results of the assay are expressed as the IC50 (the dilution of sera able to kill half of the bacteria present in the assay), represented by the reciprocal serum dilution that results in a 50% reduction of luminescence (and thus raising 50% growth inhibition). GraphPad Prism 7 software (GraphPad Software, La Jolla, CA, USA) was used for fitting and IC50 determination.

### 2.5. Statistical Analysis

Statistical analyses were performed with Minitab 18 (Minitab Inc., Chicago, IL, USA) as described in the results section. ANOVA with variance component analysis (general linear model with random factors) was used to estimate the intermediate precision (defined as the variability among different days and different operators), the repeatability (defined as the variability under the same operating conditions over a short interval of time), and to evaluate the contributions of the operator and day of analysis to the variability.

### 2.6. Ethical Statement

The human serum pool used in this study was derived from subjects enrolled in the clinical trial registered with ClinicalTrials.gov number NCT02017899. The relevant ethical and regulatory approval was obtained from the respective institutional and national ethics review committees. Written informed consent was obtained before enrollment from the subjects and the trial was designed and conducted in accordance with the Good Clinical Practice Guidelines and the Declaration of Helsinki [17].

## 3. Results

### 3.1. Development and Optimization of L-SBA on Human Sera Raised against S. sonnei GMMA 

**Figure 1:**
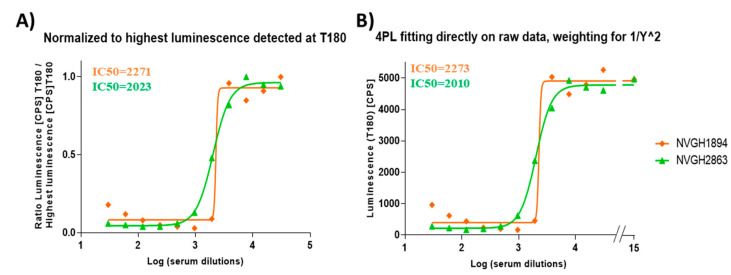
4PL fitting using different models. Representative results obtained by: (**A**) fitting performed after the normalization of luminesce raw data (counts per second (CPS)) for the highest luminescence detected in sera dilutions, as per [24]; (**B**) fitting directly to raw luminescence (CPS), adding a weighting factor of luminescence^2 in the least mean squares calculation, assigning to luminescence detected in well with no sera an arbitrary Log dilution of 15, and forcing bottom luminescence to be between 0 and 400 CPS. In the graphs, IC50 obtained in testing NVGH1894 (mouse serum) and NVGH2863 (human serum) are reported in orange and green, respectively.

In order to determine the possibility of assessing the serum bactericidal activity of human sera against *S. sonnei* by L-SBA, NVGH2863, an anti-*S. sonnei* IgG human standard serum already in use to assess the quantity of human antibodies raised upon vaccination with *S. sonnei* GMMA in clinical trials [17,18] has been used to setup the assay conditions with human sera and characterize the assay prior moving on with testing the functionality of the clinical samples. Initial experiments were conducted to test the behavior in L-SBA of NVGH2863 under experimental conditions already established with pre-clinical sera (20% exogenous baby rabbit complement and *S. sonnei* with stabilized LPS expression in vitro as target bacteria). Experiments were conducted in comparison to mouse standard serum NVGH1894, already used extensively in pre-clinical studies [24], thus serving also as bridging. Assay conditions developed in pre-clinical studies were found to be optimal also when using human sera, without the detection of prozone effect when the assaying sera finally diluted at 1:30 in the assay (Figure 1A), also confirming the non-specific killing of BRC at 20% in the assay, as previously established [24]. We have previously verified that human pre-immune sera gave no aspecific killing [26]. A prozone effect is defined for a curve readout vs. dilution (in this case luminescence vs. serum dilution) a condition in which for the first points tested (the least diluted) the readout value (luminescence) is higher than readout value obtained with points highly diluted. 

We performed an initial assessment of the homoscedasticity of the data produced at the different sera dilutions, performing 12 independent sera dilution series repeated in six different plates. The test for equal variance of the data obtained per each sera dilution (per each plate) confirmed the lack of homoscedasticity of the data (Figure S1). In the 4-Parameter Logistic (PL) fitting of luminescence vs. Log transformed sera dilutions, the sum of the squared residuals weighted for the inverse of luminescence^2 was minimized. An improved analysis method to directly obtain SBA titers from raw luminescence data was also implemented. The aim was to minimize any raw data manipulation by the operator (i.e., not to normalize for the dilution giving the highest luminescence for each dilution series, and not to individually select this value and perform calculations for each sample within each run), thus reducing to a minimum the risk of errors. The latter is a critical aspect when testing clinical samples to ensure the integrity of the data. To further improve the analysis, we also included in the 4PL fitting the luminescence value of a well with no serum by assigning to it an arbitrary Log dilution of 15 to mimic the luminescence obtained from a serum several billions time diluted, and thus representing the maximum growth of bacteria in the assay. The use of luminescence detected on the well with no serum, coupled with mathematically forcing the 4PL regression to have a bottom luminescence below the level detected with high bactericidal sera (400 counts per second), provided solid upper and lower asymptotes of the 4PL curve fitted to data, minimizing any impact of prozone (Figure 1B). Furthermore, samples with no sera represented internal controls of the assay to confirm the absence of non-specific complement-mediated killing, as well as to confirm the optimal growth of the bacteria during the assay (luminescence detected in the control wells at T180 was > 5-fold luminescence detected at T0).

With the identified assay conditions and improved analysis method, we then moved to L-SBA characterization by assessing precision (both in terms of repeatability and intermediate precision), specificity, linearity, as well as determining limit of detection and quantitation of the assay.

### 3.2. Precision

The precision of the method expresses the closeness of agreement among multiple analyses of the same homogeneous representative sample tested under the prescribed conditions. It was considered at two levels: repeatability (intra-assay variation) and reproducibility as intermediate precision (inter-assay variation). Thus, to assess precision of the assay the IC50 for NVGH2863 serum was determined independently by two operators, 12 times per day, on three different days (72 measurements in total). Log-transformed IC50s obtained by both operators on each day have been used to determine the repeatability and intermediate precision of the assay (Figure S2). 

The analysis was characterized by an intermediate precision (CV% IP) of 6.15% and a repeatability (CV% R) of 6.15%. All the variance has been in fact attributed to the repeatability: both day and operators were not significant (*p* values = 0.605 and 0.625, respectively). The average LogIC50 from all the measurements was 3.36 (or IC50 = 2528).

### 3.3. Linearity

**Figure 2:**
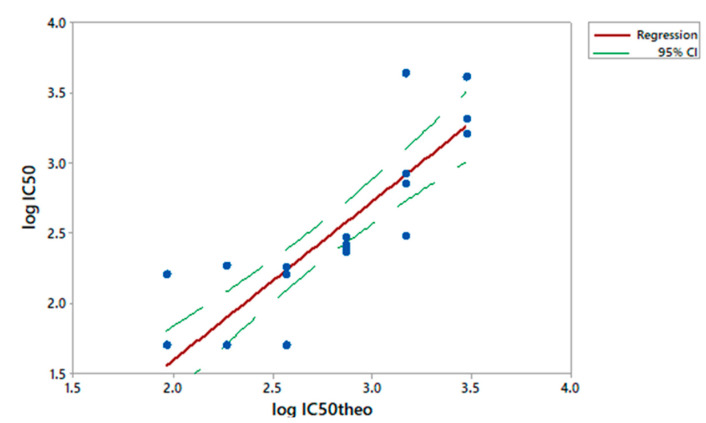
Linearity. Log(IC50 theoretical) obtained for each sample versus Log(IC50 experimentally obtained). Single datapoints are indicated with blue dots. The red solid line represents the linear regression and the green dashed line the 95% confidence interval (CI).

To assess the linearity of the assay, NVGH2863 serum was pre-diluted in PBS (neat, 2-fold, 4-fold, 8-fold, 16-fold and 32-fold times, respectively) before being probed against *S. sonnei* in L-SBA. Each pre-dilution was prepared independently by the two operators on the same day and assayed twice. We considered the average of IC50 of the undiluted serum as the “true value”, and from this one we calculated the expected IC50 based on the dilutions by volume performed (IC50 theoretical). A regression analysis was performed on Log(IC50 experimentally obtained) vs. Log(IC50 theoretical) (Figure 2). 

From the analysis of variance, the linear model was significant (*p* < 0.001) and the lack of fit not significant (*p* = 0.122) (Figure S3A). The residuals of the linear regression model were normally distributed (Figure S3B), the intercept was not significantly different from zero (95% CI: −1.412; 0.096), and the slope not significantly different from 1 (95% CI: 0.856; 1.400) (Figure S3A). 

### 3.4. Specificity

The specificity of the assay is the ability of an analytical procedure to determine solely the concentration of the analyte that it intends to measure. In case of *S. sonnei* 1790GAHB vaccine, the target antigen is considered the LPS OAg. 

We initially assessed the homologous specificity by pre-incubating homologous *S. sonnei* purified LPS at different concentrations with test serum prior to performing the L-SBA. The aim was to determine the lowest concentration of LPS able to inhibit ≥ 70% of the IC50. A homologous competitor was spiked to NVGH2863 at the final concentrations of 50, 20, 5, 1, 0.1 µg/mL; the un-depleted control was represented by NVGH2863 serum incubated with an equal volume of PBS alone. All samples were assayed in duplicate. The percentage of inhibition was determined by calculating the decrease in the observed SBA titer between samples pre-treated with competitor and un-depleted control. The lowest *S. sonnei* LPS concentration, among the ones tested, that could cause a reduction of the IC50 ≥ 70% compared to the un-depleted control sample was 0.1 µg/mL with over 90% depletion of the SBA titer. This concentration was then selected to assess the heterologous specificity. 

A second set of experiments was performed to determine the heterologous specificity. This was carried out by pre-incubating NVGH2863 serum with an equal volume of heterologous competitor at the final concentration of 0.1 µg/mL. For heterologous specificity, *S. flexneri* 1b, *S. flexneri* 2a, *S. flexneri* 3a OAg (heterologous but from the same species), and *Salmonella* Typhimurium OAg (heterologous from a different species) were tested; the internal controls for these experiments were represented by serum pre-incubated with an equal volume of PBS alone (un-depleted), and by serum preincubated with *S. sonnei* LPS (to further confirm homologous specificity). Specificity was determined as % IC50 inhibition; this was calculated using the following formula:

%IC50 inhibition = (IC50 of the un-depleted sample) − (IC50 of the sample pre-treated with competitor)/(IC50 of the un-depleted sample) * 100(1)

Depletion with 0.1 µg/mL of *S. sonnei* LPS (homologous competitor) caused an inhibition of IC50 of 95%, confirming the high specificity of the assay for *S. sonnei* LPS, whereas depletion with heterologous antigens resulted in an absent or a marginal (<30%) decrease in SBA titer, suggesting the absence of any non-*S. sonnei* polysaccharide-specific response in the assay (Table 1). 

### 3.5. Limit of Detection (LoD) and Limit of Quantitation (LoQ)

Finally, we determined the Limit of Detection (LoD) and the Limit of Quantitation (LoQ) of the assay, representing the lowest SBA titer than can be detected under the assay conditions and the lowest SBA titer that can be quantified with a suitable precision, respectively. To do so, NVGH2863 was pre-diluted in PBS to generate samples with low but detectable SBA titer. These conditions simulated the worst-case scenario possible for the assay, and thus the one expected to give the highest variability. Twelve independent serial curves were tested and IC50 calculated reported in Table 2, either starting the L-SBA at assay dilution of 1:30 (using NVGH2863 diluted 10-fold as test sample for the assay) or 1:4 (NVGH2863 diluted 100-fold as test sample for the assay).

The LoD and LoQ of the assay were calculated accordingly to the ICH guideline Q2(R1) [29], by applying the following formulas:

LoD = 10^(3.3 * SD) * X(2)

LoQ = 10^(10 * SD) * X(3)

where X represents the lowest serum dilution tested in the assay and SD represents the standard deviation of IC50 obtained for the samples. LoD and LoQ resulted to be equal to an IC50 of 45 and of 100, respectively, when the starting dilution tested in the assay was 30, or a LoD of 8 and LoQ of 33 when the starting sera dilution tested in the assay was 4. 

## 4. Discussion

The predominant readout for *Shigella* vaccine immunogenicity has been traditionally considered the serum IgG antibody level against LPS [30] that can be assessed through LPS-specific ELISA. Several assays can be considered as immunological functional readouts to determine the effectiveness of antibodies raised upon vaccination, like opsonophagocytosis or serum bactericidal assay, to determine the cell-mediated and cell-independent bactericidal activity of antibodies, respectively [20]. Although not being an established correlate of protection for *Shigella* effectiveness, the ability to cause complement-mediated killing has been assessed several times in sera both from convalescent patients and from vaccinated individuals [3,4,7]. An in vitro assay to assess complement-mediated killing represents a key indication of the functional activity of antibodies raised upon vaccination with *Shigella* vaccine candidates, and this assay is traditionally represented by SBA. The traditional SBA method used to determine the bactericidal activity of *Shigella* sera from clinical trials relies on a laborious process of plating bacteria on solid media, overnight incubation, colony counting [31], and end-point titer calculation without an interpolation of all sera dilutions tested [8]. This method is therefore often time consuming, highly variable, operator dependent and thus difficult to perform with consistency (Table 3). 

To overcome these limitations, we have recently developed an high-throughput SBA method based on luminescence readout (L-SBA), that has been already extensively described, to determine the level of functional antibodies in vitro [24]. In this study, we have presented the further optimization and characterization of L-SBA on human sera. The GSK Vaccines Institute for Global Health (GVGH) is working on developing a multivalent vaccine against *Shigella* based on GMMA technology. The most advanced *S. sonnei* component (1790GAHB) has been tested in phase I and phase II clinical trials. Immunogenicity has been evaluated so far in terms of anti-LPS IgG response induced [17,18,19]. The work performed here will allow the analysis of the clinical samples by L-SBA, confirming whether the antibodies elicited by 1790GAHB are able to kill *Shigella* (i.e., functionality of antibodies induced). Our L-SBA method will help to better study the immunogenicity profile of vaccines against *Shigella* also helping to establish correlation between SBA and ELISA titers and in the long term to establish a correlate of protection still not identified. The method presented here will also allow us to analyze bactericidal activity in sera from convalescent patients and therefore eventually define an SBA titer representing the threshold for protection against *Shigella* infections.

We have successfully optimized the fitting of the data, including in data analysis a point mimicking a sera billion times diluted, allowing us to establish conditions in which, without any normalization, SBA titers can be directly obtained from raw luminescence values. The latter represents a crucial aspect to increase the throughput of the assay, but especially to reduce any potential bias due to the manipulation of raw data when testing clinical samples. 

In this work, we have characterized L-SBA on human sera, demonstrating that in the working conditions tested, it is able to detect sera having a SBA titer as little as 8, with virtually no upper limit of detection, and to quantify with precision sera with IC50 of 33. The assay showed low variability, in particular the repeatability corresponding to the intermediate precision (CV% of 6.15%). Neither the operator nor day of analysis were significant to the overall variability. Furthermore, L-SBA was highly specific for the key active ingredient of the vaccine candidate, as, by depleting the serum with as little as 0.1 µg/mL of homologous LPS, more than a 95% reduction of IC50 was observed, whereas no depletion was observed when depleting sera with *S. flexneri* 2a, *S. flexneri* 3a and *Salmonella* Typhimurium OAg, with only a marginal SBA titer depletion (28%) observed after incubation with *S. flexneri* 1b OAg. The linearity of the assay was also assessed, and it was found to be good within the tested range with a slight deflection with more diluted samples, as reported for similar assays [23]. In line with that, good fitting of the data was obtained with a second order exponential model (Figure S4).

Using the SBA configuration described here, up to 132 specimens can be tested per day by a single operator, including a standard serum to validate each plate (12 plates can be assayed per operator per day). The analytical throughput of the described *Shigella* SBA is superior to that of another high-throughput assay recently described by Nham et al. [23], not only in terms of the number of samples that can be assayed by one operator in one day, but also in terms of not having to rely on the overnight incubation of plates to enable the bacteria to grow and become colonies (Table 3). As our assay uses standard reagents and requires only a luminometer to detect ATP, L-SBA can be considered simple enough to be adopted by laboratories around the world. Although inter-laboratory variability was not evaluated in our study, in case this would be observed, results could be normalized with the use of reference serum [23]. 

In conclusion, L-SBA applied to human sera represents an assay fully suitable to perform clinical analysis in high-throughput. Due to its specificity and versatility, L-SBA can be applied to determine the bactericidal activity of clinical sera raised against different *Shigella* serotypes, helping the development of vaccines not only in single component but also when they are in multi-component formulations. Our L-SBA method can be easily extended to other pathogens, as the method has been already demonstrated to have a similar performance against a broad range of pathogens using animal samples [24,26].

## Figures and Tables

**Table 1:** Specificity determination. Depleted samples were spiked with 0.1 µg/mL of homologous or heterologous purified polysaccharide; un-depleted samples were spiked with PBS only.

Samples Assayed		IC50	Average IC50	% IC50 Inhibition
**Un-depleted**		922	890	-
		859		
**Homologous specificity**	**Depleted with *S. sonnei*** **LPS**	39	46	**95**
		54		
**Heterologous specificity**	**Depleted with *S. flexneri* 1b OAg**	665	654	**27**
		644		
	**Depleted with *S. flexneri* 2a OAg**	912	1253	**0**
		1594		
	**Depleted with *S. flexneri* 3a OAg**	1205	1210	**0**
		1215		
	**Depleted with *S.* Typhimurium OAg**	1668	1605	**0**
		1543		

**Table 2:** Determination of limits of detection and quantitation of the assay. IC50 obtained in samples with low SBA titer.

Repeat	1	2	3	4	5	6	7	8	9	10	11	12	**Average**	**SD**
**IC50 determined starting assay at 1:30 dilution**	109	112	92	89	93	91	94	119	89	118	90	84	104	8.9
**IC50 determined starting assay at 1:4 dilution**	10	7	9	9	11	11	9	13	13	14	11	13	11	2.1

**Table 3:** Evaluation of throughput between traditional and high-throughput luminescence-based SBA (L-SBA).

Method	L-SBA	Traditional CFU-Based SBA
**Total time of execution**	7 h ^1^	1.5 working day ^2^
**Data acquisition**	2 min/SBA plate	2–3 h/SBA plate ^2^
**Reproducibility**	Higher operator independency	Lower operator independency
**Assay throughput**	1 operator/day: 132 individual sera in single (12 SBA plates total)	1 operator/1.5 day: 12 individual sera in single (1 SBA plate ^2^)

^1^ To execute one set of 12 SBA plates; ^2^ to execute one SBA plate, plating each reaction well in one full agar plate: one SBA plate corresponds to 96 agar plates. CFU = Colony Forming Unit.

## Supplementary Materials

The following are available online at https://www.mdpi.com/2571-5135/9/2/14/s1, Figure S1: Test for equal variances for homoscedasticity. Variances (x axis) from 12 replicates versus Log sera dilutions (y axis) were plotted for each of 6 different plates tested. Figure S2: ANOVA with variance component analysis obtained from the 72 individual LogIC50 produced by two operators in 3 different days, each day assaying independently twelve times the same sera. Figure S3: (A) Regression analysis for linearity assessment; (B) Residual plots for LogIC50. Figure S4: Linearity.
