# Supplementary material for: Intra-Laboratory Evaluation of Luminescence Based High-Throughput Serum Bactericidal Assay (L-SBA) to Determine Bactericidal Activity of Human Sera against Shigella
Source: High Throughput. 2020 Jun 8;9(2):14. doi: 10.3390/ht9020014 (PMC7361673; doi:10.3390/ht9020014)
Supplement: Supplementary file 1 [file high-throughput-09-00014-s001.pdf]

Supplementary Materials: Intralaboratory Evaluation of Luminescence Based High-Throughput Serum Bactericidal Assay (L-SBA) to Determine Bactericidal Activity of Human Sera against *Shigella*

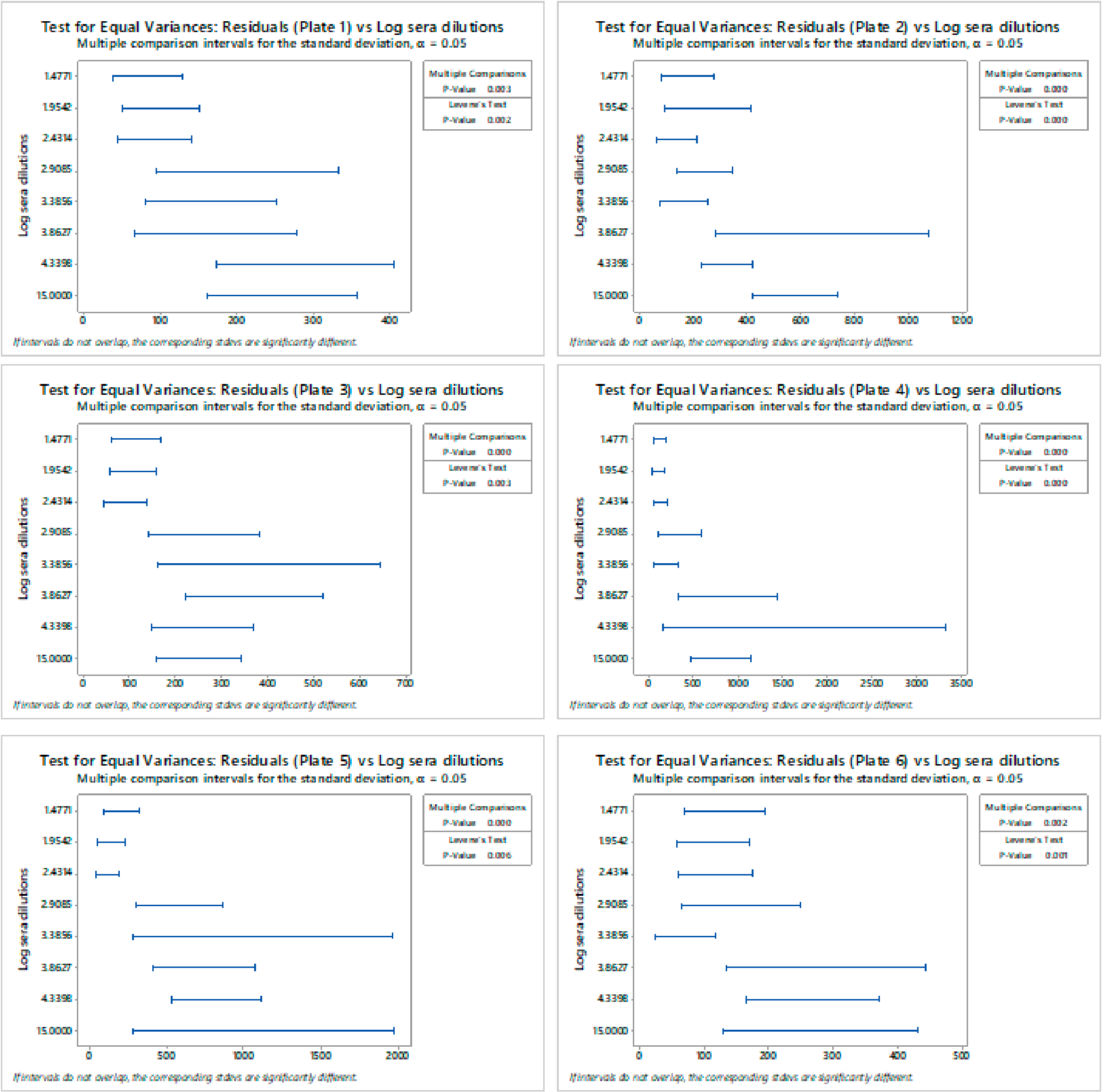

Figure S1: Test for equal variances for homoscedasticity. Variances (x axis) from 12 replicates versus Log sera dilutions (y axis) were plotted for each of 6 different plates tested.

General Linear Model: LogIC50 versus Day; Operator

Method

Factor coding (-1; 0; +1)

Analysis of Variance

Source	DF	Adj SS	Adj MS	F-Value	P-Value
Day	2	0.04307	0.02154	0.51	0.605
Operator	1	0.01025	0.01025	0.24	0.625
Error	68	2.89545	0.04258		
Lack-of-Fit	2	0.52420	0.26210	7.30	0.001
Pure Error	66	2.37125	0.03593		
Total	71	2.94877			

Variance Components, using Adjusted SS

Source	Variance	% of Total	StDev	% of Total
Day	-0.000876847*	0.00%	0.000000	0.00%
Operator	-0.000898176*	0.00%	0.000000	0.00%
Error	0.0425802	100.00%	0.206350	100.00%
Total	0.0425802		0.206350	

* *Value is negative, and is estimated by zero.*

Figure S2: ANOVA with variance component analysis obtained from the 72 individual LogIC50 produced by two operators in 3 different days, each day assaying independently twelve times the same sera.

A)

Regression Analysis: log ic50 versus log ic50theo

Analysis of Variance

Source	DF	Seq SS	Contribution	Adj SS	Adj MS	F-Value	P-Value
Regression	1	8.0709	77.02%	8.0709	8.07088	73.73	0.000
log ic50theo	1	8.0709	77.02%	8.0709	8.07088	73.73	0.000
Error	22	2.4084	22.98%	2.4084	0.10947		
Lack-of-Fit	5	0.9071	8.66%	0.9071	0.18143	2.05	0.122
Pure Error	17	1.5013	14.33%	1.5013	0.08831		
Total	23	10.4793	100.00%				

Model Summary

S	R-sq	R-sq(adj)	PRESS	R-sq(pred)
0.330866	77.02%	75.97%	2.87532	72.56%

Coefficients

Term	Coef	SE Coef	95% CI	T-Value	P-Value	VIF
Constant	-0.658	0.364	(-1.412; 0.096)	-1.81	0.084	
log ic50theo	1.128	0.131	(0.856; 1.400)	8.59	0.000	1.00

B)

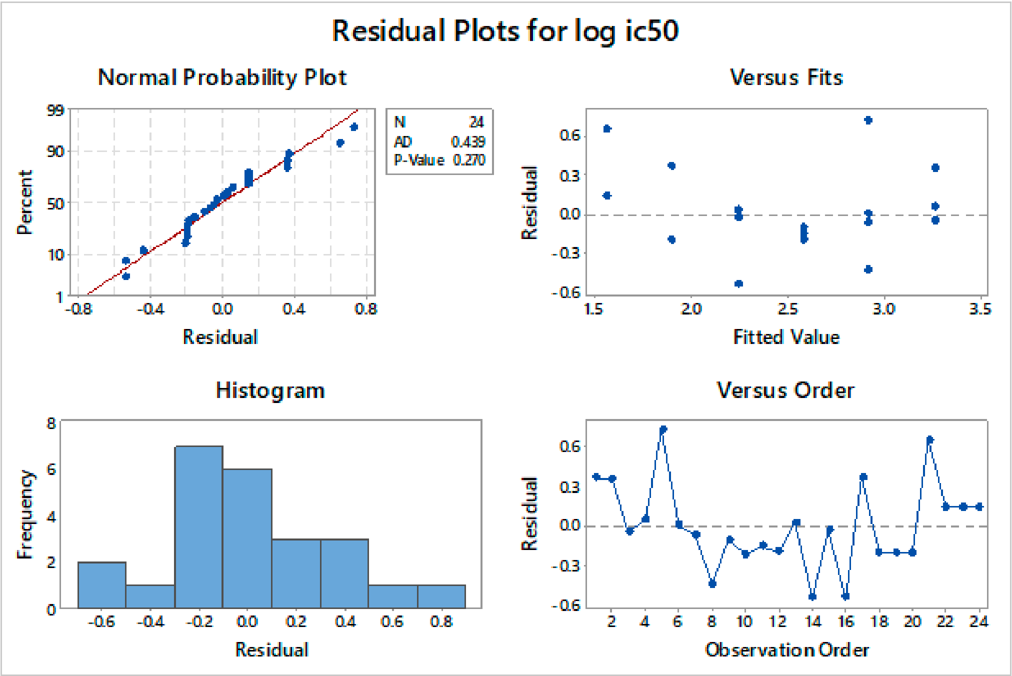

Figure S3: (A) Regression analysis for linearity assessment. (B) Residual plots for LogIC50.

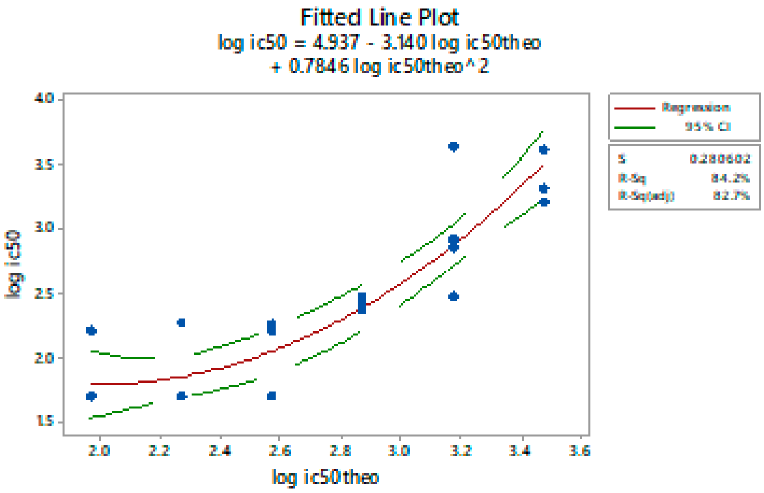

Figure S4: Linearity. Log(IC50 theoretical) obtained for each sample versus Log(IC50 observed) are reported. Single datapoints are indicated with blue dots. Red solid line represents second order exponential regression and green dashed line the 95% confidence interval (CI).
